# Long-term physical activity: an exogenous risk factor for sporadic amyotrophic lateral sclerosis?

**DOI:** 10.3109/21678421.2016.1154575

**Published:** 2016-03-21

**Authors:** Ceryl A. Harwood, Kate Westgate, Sue Gunstone, Soren Brage, Nicholas J. Wareham, Christopher J. McDermott, Pamela J. Shaw

**Affiliations:** ^a^ Academic Neurology Unit, Sheffield Institute for Translational Neuroscience (SITraN), Department of Neuroscience, Faculty of Medicine, Dentistry and Health, University of Sheffield, 385A Glossop Road, Sheffield, S10 2HQ, UK; ^b^ Medical Research Council Epidemiology Unit, University of Cambridge, Box 285 Institute of Metabolic Science, Cambridge Biomedical Campus, Cambridge, CB22 0QQ, UK

**Keywords:** Amyotrophic lateral sclerosis, Physical Activity, Risk factor, Epidemiology, Gene-environment interaction

## Abstract

*Objectives*: To conduct a geographically defined, UK-based case-control study, to examine any association between physical activity (PA) and amyotrophic lateral sclerosis (ALS). *Methods*: A novel historical PA questionnaire was designed, validated, and subsequently administered in individual face-to-face interviews of 175 newly diagnosed sporadic ALS cases and 317 age- and sex-matched community controls. Historical PA energy expenditure and time spent in vigorous-intensity PA were derived from questionnaire data and compared between cases and controls. *Results*: Participation in an extra 10kJ/kg/day of PA (equivalent to approximately 45minutes brisk walking) was consistently associated with an increased risk of ALS, with the strongest association observed for adulthood exercise-related PA (OR 1.47, 95% CI 1.10-1.97). An extra 10mins/day of vigorous PA was also associated with the odds of ALS (OR 1.03, 95% CI 1·01-1·05). Results were slightly attenuated following adjustment for smoking and educational attainment. *Conclusions*: To our knowledge this is the first study to demonstrate a positive association between ALS and PA participation using a specifically designed and validated historical PA questionnaire. Despite the well-established health benefits of PA, a high activity lifestyle may also be associated with elevated risk of ALS. Large-scale prospective studies in the future may help to confirm this association.

## Introduction

Amyotrophic lateral sclerosis (ALS) is a disabling, terminal neurodegenerative condition characterised by progressive paralysis and atrophy of limb, bulbar and ultimately respiratory muscles. Population-based research demonstrates geographical variation in incidence, with an annual incidence in Europe and North America of approximately 2 per 100,000 (1,2) and lower incidence suggested in African, Asian and Hispanic populations (3).

Epidemiological, biological and genetic research suggests a complex pathogenic interaction between genetic and environmental exposures (4). However, aside from inherent risk associated with genotype, age and gender, very few exogenous causative factors have been confirmed. The strongest evidence relates to smoking, which was associated with incident ALS in a recent meta-analysis of prospective studies including over 1 million participants (5). Following proposals of an association between physical activity (PA) and the development of ALS (6), several observational studies have examined this hypothesis, with inconsistent results (7–11). Reports of elevated ALS incidence in Italian professional footballers stimulated further epidemiological research (12–14). However, methodological challenges when examining exogenous associations with relatively rare diseases, particularly regarding sample size, bias minimisation, and valid exposure quantification, have limited the conclusions drawn, though recent studies have improved on some of these concerns (15,16).

Beyond epidemiology, it has been proposed that elevated tissue metabolism and motor neuron firing during exercise augment oxidative stress and glutamate excitotoxicity-related motor neuron degeneration (17,18). Several genes associated with ALS are also known to be up-regulated by PA (19,20). Homeostatic abnormalities in ALS which may respond to PA participation provide an alternative mechanism for a disease association (21).

We hypothesised that participation in a high level of PA is associated with an increased risk of sporadic ALS. Our objectives were to conduct a case-control study, recruiting a UK-based study population, to determine any association between PA and ALS.

## Materials and Methods

ALS patients and community controls were recruited between July 2009 and July 2013 from a geographically defined population within Northern England comprising approximately 7·6 million individuals. Relevant NHS and university research ethical approvals were obtained and procedures followed were in accordance with these standards. All study participants provided informed consent.

### Study population

Individuals with clinically definite, probable or possible sporadic ALS (22), were identified from hospital and community ALS services. Patients were eligible if recruited within 6 months of diagnosis to ensure near-incident rather than long-prevalent cases. Those with any family history of ALS or concurrent neurodegenerative disease were excluded. Potential controls were identified from a selection of general medical practices within the same regions, covering a broad spectrum of patient demographics. To recruit two controls per case, 10 individuals without an active neurological diagnosis, and matched to each case by date of birth (+/-5 years) and gender, were randomly selected for invitation by their general practitioners. All eligible participants were sent written information about the study, and individual appointments arranged with those who decided to participate.

### Data collection

Face-to-face interviews, lasting approximately 90 minutes, were conducted with each participant by one of two interviewers, using a standardised approach. PA data were collected using a novel, valid electronic questionnaire, the Historical Adulthood Physical Activity Questionnaire (HAPAQ)(23). In brief, HAPAQ collects data regarding total adulthood PA at home, work and in leisure (exertional sport and casual exercise). Closed questions regarding the nature, frequency and duration of activities are asked in periods of 5 or 10 years, using a life calendar of significant events to aid recall. Participants were blinded to the research hypothesis.

### Data reduction from the questionnaire

To quantify PA volume, PA energy expenditure (PAEE) scores for each activity were derived from questionnaire data using the Compendium of Physical Activities (24). This resource quantifies the energy expenditure of activities as a ratio with standard resting metabolic rate, expressed as metabolic equivalents (METs), with one MET equivalent to an approximate oxygen consumption of 3·5ml O_2_/kg/min. MET intensity values for each reported activity were identified from the Compendium, subtracted by 1 MET for resting metabolic rate, and then multiplied by the reported activity durations. The average daily PAEE (kJ/kg/day) was calculated using the following equation, assuming the energy equivalent of one litre of oxygen to be 20·3kJ (25):

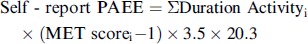



Individual average daily PAEEs from occupational, leisure and total PA were summarised for the most recent 15 years, reflecting the period HAPAQ was validated, and for the whole of adulthood. The time spent in vigorous PA, defined as intensity ≥6.0 METs, was also determined (26). Data for the most recent five years were excluded to limit PA suppression which might result from subclinical disease. When calculating total volume of PA, housework-related activity was excluded, following observations of an inverse association of self-reported housework with objectively assessed PAEE (27).

### Statistical analysis

To detect an odds ratio (OR) of 1.8, at 80% power and an alpha value of 0.05, 175 cases and 350 controls were required to compare participants in the highest quartile of total PAEE with those below the 75th centile.

STATA/IC version 13·0 statistical package was used for the analyses. Baseline participant characteristics were tabulated by case status. Separate conditional logistic regression models determined the independent ORs for the association between ALS and several PA exposure variables: average daily total PA, time spent in vigorous PA, and activity during leisure and work. Adjustment was made for age as a continuous variable based on knowledge of greater ALS risk with increasing age, evidence of a negative correlation between age and PA in the study population, and plausibility of a confounding role. As evidence regarding the association of smoking with ALS remains inconclusive, we present both adjusted and unadjusted models with regards to smoking (5,28). Further adjustment was made for educational attainment (school only or professional/vocational qualification or university qualification). We also examined the association between age of onset of ALS (cases) or recruitment (controls) and PA participation using a linear regression model, formally tested using an interaction term between PA and case status.

## Results

**Figure 1.**
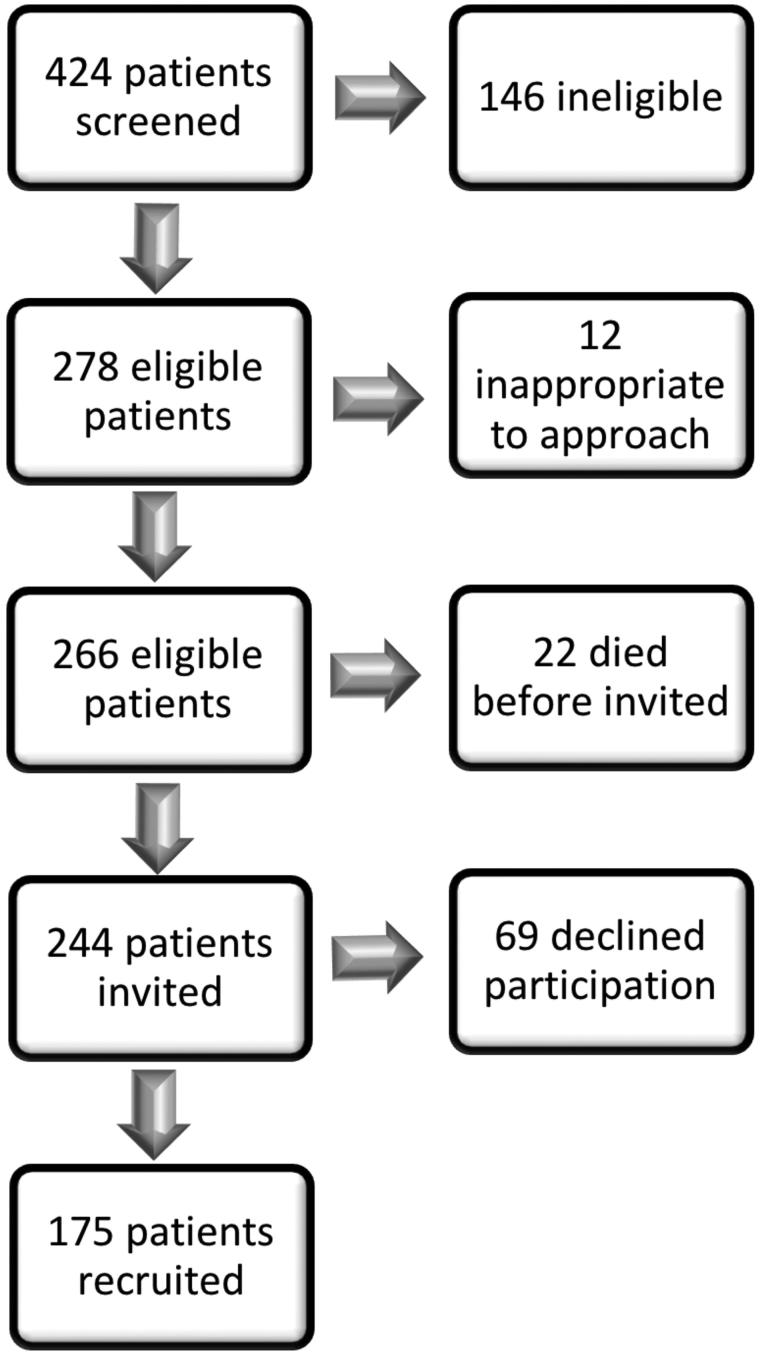
Selection process for recruitment.

424 MND patients were screened for the study. Figure 1 describes the case selection process. Of those screened, 146 patients did not meet the eligibility criteria or lived outside the recruitment area. 34 eligible patients were too unwell or emotionally distressed to approach regarding research participation. From the remaining 244 patients, 175 were recruited. Regarding controls, all those agreeing to participate and able to attend an interview within the study timeframe, were recruited. Every consented participant completed the data collection process, with no missing data for any variables.

Table 1 displays the baseline characteristics of the participants. 68% of cases had clinically definite or probable ALS. Almost 70% of cases presented with limb-onset disease and 28% with bulbar-onset. Cases and controls were similar regarding age, gender, smoking status and education. Within the most recent 15 years, 28.3% of controls and 30.1% of ALS cases had retired from work.

**Table 1.** Demographic and clinical characteristics of study participants. Quantitative values are given as medians (interquartile range) or n (%).

Variable	Controls (n = 317)	Cases (n = 175)
Basic demographics		
Number of males (%)	197 (62·2)	109 (62·3)
Age (years)	65 (full range 36-91)	64 (full range 26-88)
Retired in last 15 years, n (%)	269 (28·3)	158 (30·1)
El-Escorial diagnostic criteria		
Clinically definite, n (%)		45 (25·7)
Clinically probable, n (%)		74 (42·3)
Clinically probable lab-supported, n(%)		43 (24·6)
Clinically possible, n (%)		13 (7·4)
Site of onset		
Lower limb, n (%)		57 (32·6)
Upper limb, n (%)		65 (37·1)
Bulbar, n (%)		49 (28·0)
Respiratory, n (%)		4 (2·3)
Smoking status		
Non-smoker, n (%)	86 (27·1)	50 (28·6)
Ex-smoker, n (%)	199 (62·8)	108 (61·7)
Current smoker, n (%)	32 (10·1)	17 (9·7)
Education		
Age left fulltime education (years)	16 (full range 12-35)	16 (full range 12-25)
School education only, n (%)	90 (28·4)	62 (35·4)
Professional/vocational qualification, n (%)	147 (46·4)	84 (48·0)
University qualification, n (%)	80 (25.2)	29 (16.6)

**Figure 2.**
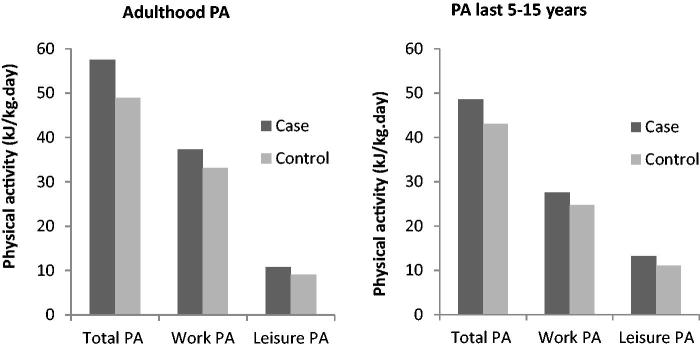
Self-reported median daily physical activity (PA) exposure (kj/kg/day) by outcome.

ALS cases consistently reported higher total PA, time spent in vigorous PA and PA in work and leisure compared to controls (Table 2 and Figure 2). However, the range of reported PA was wide and overlapping for both sets of participants. Regarding domain-specific activity, 33·4% controls and 30·9% cases reported no adulthood sport participation, reflected by the lower limit of the related interquartile ranges in Table 2. When comparing the two time periods, 44·8% of controls and 42·9% of cases reported no vigorous PA during the last 15 years compared to 20·2% and 14·3% respectively throughout the whole of adulthood.

**Table 2.** Self-reported physical activity (PA) exposure by participants.

Physical activity (PA) variable	Controls (n = 317)		Cases (n = 175)	
	Median	IQR	Median	IQR
Average daily PA during the last 5–15 years				
Total PA (kJ/kg/day)	43·1	27·1–68·6	48·6	27·7–76·0
Time spent in PA > 6METs (mins/day)	2.4	0·0–24.0	4.2	0·0–36.0
Work related PA (kJ/kg/day)	24·7	11·9–46·7	27·5	9·6–54·9
Total leisure PA (kJ/kg/day)	11·0	4·0–21·0	13·2	4·6–27·5
Exertional sport related PA# (kJ/kg/day)	0·0	0·0–7·3	0·0	0·0–9·8
Casual exercise related PA+ (kJ/kg/day)	5·5	2·2–12·2	8·4	3·1–15·9
Average daily adulthood PA				
Total PA (kJ/kg/day)	48·9	32·6–71·6	57·5	35·2–98·7
Time spent in PA > 6METs (mins/day)	9.6	0·6–30.6	13.2	1.2–74·4
Work related PA (kJ/kg/day)	33·1	21·5–56·5	37·3	22·5–74·3
Total leisure PA (kJ/kg/day)	9·1	4·0–18·2	10·8	4·6–22·3
Exertional sport related PA (kJ/kg/day)	2·2	0·0–9·6	2·4	0·0–11·0
Casual exercise related PA (kJ/kg/day)	4·3	1·8–8·7	5·7	2·5–10·5

IQR: Interquartile range

# Exertional sport related PA: sporting activities causing sweating and breathlessness

+ Casual exercise related PA: regular gentle exercise without causing sweating and breathlessness

Table 3 reports the ORs regarding the association between PA participation and ALS status. A difference in daily PAEE of 10kJ/kg, equivalent to approximately 45 minutes of brisk walking or 20 minutes of soccer, was consistently associated with an increase in the odds of having ALS. Significantly positive associations were demonstrated for both time periods and across most PA domains. The strongest association related to participation in higher levels of adulthood casual exercise PA (age adjusted OR 1·47, 95% CI 1·10-1·97, p = 0.01). Total PA throughout adulthood was also significantly associated with ALS (age adjusted OR 1·09, 95% CI 1·03–1·16, p = 0.002), as was undertaking an extra 10 minutes of vigorous PA per day (age adjusted OR 1·03, 95% CI 1·01–1·05, p = 0.004). Further adjustment for both smoking and educational attainment resulted in only very minor attenuation of the observed size of effects. Adulthood exertional sport related activity and work related activity within the most recent 15 years were not significantly associated with ALS, possibly reflecting a reduction in power from the smaller domain-specific sample sizes described above.

**Table 3.** Odds ratios for the association of physical activity (PA) with MND per unit change of 10 kJ/kg/day of PA or 10 mins/day of time spent in vigorous PA.

Physical activity (PA) variable	Odds ratio* (95% CI)	p value	Odds ratio† (95% CI)	p value
Average daily PA in last 5–15 years (kJ/kg/day)				
Total PA	1·05 (1·00–1·11)	0·038	1.04 (0.99–1.10)	0.084
Time spent in PA >6METs (mins/day)	1·02 (1.00–1·04)	0·071	1.01 (0.99–1.03)	0.141
Work related PA	1·03 (0·98–1·09)	0·259	1.02 (0.97–1.08)	0.434
Total leisure PA	1·19 (1·05–1·34)	0·006	1.19 (1.04–1.35)	0.008
Exertional sport related PA	1·12 (0·97–1·29)	0·132	1.12 (0.97–1.31)	0.131
Casual exercise related PA	1·30 (1·08–1·57)	0·007	1.28 (1.06–1.55)	0.011
Average daily adulthood PA (kJ/kg/day)				
Total PA	1·09 (1·03–1·16)	0·002	1.08 (1.02–1.15)	0.009
Time spent in PA >6METs (mins/day)	1·03 (1·01–1·05)	0·004	1.03 (1.01–1.05)	0.013
Work related PA	1·09 (1·03–1·16)	0·006	1.08 (1.01–1.15)	0.021
Total leisure PA	1·17 (1·00–1·37)	0·056	1.15 (0.98–1.36)	0.089
Exertional sport related PA	1·06 (0·88–1·27)	0·539	1.04 (0.87–1.26)	0.660
Casual exercise related PA	1·47 (1·10–1·97)	0·010	1.45 (1.08–1.96)	0.014

* Adjusted for age as a continuous variable

† Adjusted for smoking and education as categorical variables

An earlier age of disease onset was observed with increasing adulthood PA participation. Age of onset was 0·34 years (95% CI −0·677 to −0·002) lower for every additional 10kJ/kg/day of total PAEE (p = 0·049) and 0·64 years (95% CI -1·392 to 0·112) lower for an extra hour of vigorous PA daily (p = 0·095). In comparison, age at recruitment of the controls was 0.43 years (95% CI −0.728 to −0.124) lower for every additional 10kJ/kg/day of total PAEE (p = 0·006) and 0.86 years (95% CI −1.584 to −0.134) lower for an extra hour of vigorous PA daily (p = 0·020). There was no significant interaction of the effects of age on PA by case status (p = 0.719).

## Discussion

To the best of our knowledge, this is the first study to use a specifically designed and validated PA questionnaire to demonstrate a positive association between PA and the development of sporadic ALS. Positive associations were reported for all domains of PA and both time periods, and remained following adjustment for potential confounders, with the strongest associations for those reporting higher levels of exercise-related PA throughout adulthood. However, our results do not support an earlier age of onset of ALS with greater PA participation as a similar age-related association was also demonstrated within the control group, reflecting a universal decline in PA participation with increasing age.

Our findings are in agreement with several published studies. ALS has been reported as associated with participation in various domains of PA, including soccer, leisure activities and varsity athletics (8,12,14,29). Conversely, some studies have reported no association (7,9,10). Regardless of the findings, all the previous studies have methodological limitations, particularly relating to recruitment strategy and the quality of the exposure data, restricting the conclusions which can be drawn. Interestingly, a difference in risk between soccer (elevated) and other sports such as cycling and basketball (lower) has been described, resulting in proposals of soccer-specific risk factors rather than PA participation per se, though small cohort sample sizes may also explain these observations (30).

Contrasting results have been reported from two recent case-control studies: a Dutch study, demonstrating a positive association between ALS and leisure PA but no other associations, and a European study, reporting a potential protective effect of PA relating to the development of ALS (15,16). Strengths of these studies include population-based recruitment and large sample sizes. However, both studies reported either missing data or proxy informants for around 18% participants, blinding procedures were not described in the European study, and neither study described validity of the exposure assessment, making interpretation of their results more difficult.

A recent systematic review of epidemiological studies examining the association between PA and ALS concluded that PA was not a risk factor, but was inconclusive regarding the risk associated with occupational activity and soccer (31). Although useful criteria were applied to classify the reviewed evidence, these criteria do not consider the validity of exposure quantification, a key element in determining data quality (32). The review also acknowledged significant variation in PA quantification methodology and resultant accuracy, suggesting this may contribute to inconsistent findings, such as the contrasting conclusions in the two recent studies (15,16). The use of a validated instrument in the current study goes some way towards addressing this limitation (23), although it should be noted that lack of validation of other instruments does not necessarily imply poor validity.

Geographically-defined variations in genetic susceptibility and exposure status may also contribute to reported discrepancies between studies. Differences in ALS susceptibility gene mutations between populations are recognised, as illustrated by variable prevalence of superoxide dismutase-1 (SOD1) mutations in different countries (33). Work, leisure and transport-related PA patterns are also likely to differ between countries, raising questions regarding whether relative PA participation or a threshold level is aetiologically relevant.

The reported associations with total PAEE in the present study suggest that overall cumulative PA participation may be relevant in the pathogenesis of ALS. Any associations between ALS and PA most likely represent interactions between genetically determined susceptibility and exposure to potentially modifiable lifestyle behaviours. Various mechanisms have been proposed to explain such a gene-environment interaction. As normal physiological responses to PA may interact with several pathogenic mechanisms relevant to ALS, exercise might act as a trigger for ALS, with neurodegenerative consequences in those susceptible to the effects. Alternatively, individuals may be unable to mount the normal physiological protective responses to PA, such as the compensatory rise in antioxidant capacity (34), due to genetic variations which simultaneously contribute to the pathogenesis of ALS. A particular genetic profile may also contribute to sporting ability and ALS propensity (35), simultaneously increasing ALS risk and PA participation. This would be consistent with reports of lower weight and reduced cardiovascular morbidity in ALS patients, which may confound this ALS association (8,36). Also, hypermetabolism has been demonstrated in ALS, which could aetiologically link this disease and PA through exacerbation of pathogenic abnormalities in homeostasis or a simultaneous predisposition to ALS and PA, though conversely could just represent the consequences of activity participation (21,37). Further work is needed to determine the mechanisms of this potential indirect association, particularly the timing of homeostatic variations in relation to disease onset.

The recent work by Huisman, Pupillo and Hamidou represent important contributions to understanding associations between ALS and PA, but may lead some to conclude that this research question has already been answered (15,16,31). However, all previous studies on this topic, including the current one, highlight the challenges of a case-control study design imposed by the rarity of ALS, but we propose that this report represents a rational approach when examining environmental exposure associations with rare diseases of unknown latency period. We chose to focus on using a specifically designed and validated questionnaire, able to rank individuals according to their activity levels (23). Although alternative PA questionnaires exist, few measure historical PA, with HAPAQ being the first to be validated against repeated historical objective activity measurements. Administration of HAPAQ through individual, standardised face-to-face interviews, using a life calendar based on key life events to aid recall, facilitated complete, accurate data collection. This represents a unique approach not previously reported in other studies on this topic, contrasting with methodology using non-validated postal questionnaires, whose main advantage is the ability to achieve large recruitment samples. Other strengths of the present study include clear case definition using established diagnostic criteria, and the recruitment of controls from a similar general practice patient source from which ALS cases were derived.

Interpretation of the results should be made whilst considering potential limitations of this study. As the role of C9ORF72 mutations in ALS pathogenesis had not been recognised at the time this study was initiated, participant genotyping was not routinely undertaken as the percentage of sporadic ALS cases with mutations in known susceptibility genes was estimated to be minimal. However, DNA samples were collected and stored from the majority of study participants, thus facilitating future examination of potential gene-environment interactions.

Retrospective data collection risks differential recall of exposures, with cases potentially overestimating activity levels, particularly if they were aware of the proposed association. To minimise this, all participants were blinded to the research hypothesis and uncertainty regarding any association between ALS and PA was made clear during each interview. The social desirability of exercise makes PA overestimation a possibility, potentially biasing the result towards the null if it was non-differential by case status, but could bias results in either direction if it was not. The previously reported under- and over-reporting of PA in active and more sedentary individuals, respectively, by HAPAQ would have similar effects (23).

The focus on PA and the social desirability of this behaviour may have attracted more active controls to participate in the study, risking selection bias. However, this direction of bias would reduce the size of the disease association detected. An acceptable recruitment rate was achieved (71.7%) from invited cases, though case ascertainment for the geographically defined recruitment population was incomplete. Although only partial data were available regarding eligible cases who declined participation, characteristics of recruited cases were comparable to the general ALS population, suggesting a representative case sample. Although interviewers were not blinded to the participants’ disease status, HAPAQ was administered in a structured manner dictated by time periods, ensuring standardised data collection for all participants and minimising interviewer bias.

HAPAQ was validated using a mixed gender population of comparable average age to the participants in the present study. However, participants in the validation sample were not defined by a neurological disease such as ALS and it is possible that ALS patients may recall PA differently. Also, HAPAQ validity was established only for the most recent 15 years. However, this does represent a historical timeframe and identical questions were repeated throughout each questionnaire time period. A prospective cohort study design with very long follow-up time and repeated assessment of PA before occurrence of ALS would eliminate many of the limitations already discussed. However, given the rarity of ALS, very few existing cohort studies will have the potential to answer this research question.

In summary, this study suggests a positive association between PA and the development of sporadic ALS using validated methodology. Although ORs approximate percentage risk with rare diseases, the contribution of PA to the risk of ALS within this study population can only be quantified by understanding the relevant gene-environment interaction. With individual risk likely to be dictated by inherent genetic predisposition, identifying this susceptibility is imperative if advice regarding lifestyle modification is to be given, particularly given the established health benefits of PA (38,39). Establishing the genetic interaction would clarify whether PA represents a direct ALS risk factor or a proxy for one or more specific genotypes through which propensity to athleticism and ALS are both expressed.

## Supplementary Material

ALS_statistician_statement_Dec_2015__1_.pdf
