# Supplementary material for: Long-term physical activity: an exogenous risk factor for sporadic amyotrophic lateral sclerosis?
Source: Amyotroph Lateral Scler Frontotemporal Degener. 2016 Mar 21;17(5-6):377–84. doi: 10.3109/21678421.2016.1154575 (PMC4950417; doi:10.3109/21678421.2016.1154575)
Supplement: ALS_statistician_statement_Dec_2015__1_.pdf [file iafd_a_1154575_sm3060.pdf]

UNIVERSITY OF CAMBRIDGE

MRC | Epidemiology Unit

Editor
Amyotrophic Lateral Sclerosis and Frontotemporal Degeneration

3 December 2015

Dear Sir/Madam

Harwood et al. Long-term physical activity: an exogenous risk factor for sporadic amyotrophic lateral sclerosis?

I confirm that the analyses presented in this paper use valid statistical methods.

Yours faithfully

Stephen J. Sharp

Stephen Sharp, MA MSc
Senior Statistician

MRC Epidemiology Unit
University of Cambridge School of Clinical Medicine
Box 285, Institute of Metabolic Science
Cambridge Biomedical Campus, Cambridge, CB2 0QQ
Tel: +44 (0) 1223 330 315 Fax: +44 (0) 1223 330 316
www.mrc-epid.cam.ac.uk
